# Surgeon-General Sternberg

**Published:** 1898-03

**Authors:** 


Surgeon-General Sternberg, of the United States Army, has been elected President of the American Medical Association for the coming year.

